# Statin Therapy in Patients Undergoing Thoracic Aorta Replacement for Aortic Aneurysms

**DOI:** 10.1055/s-0041-1730296

**Published:** 2021-11-08

**Authors:** Bogdan A. Kindzelski, Andrea L. Hanick, Kyle G. Miletic, Ashley M. Lowry, David Van Wagoner, Eugene H. Blackstone, Eric E. Roselli

**Affiliations:** 1 Aorta Center, Department of Thoracic and Cardiovascular Surgery, Heart, Vascular, and Thoracic Institute, Cleveland Clinic, Cleveland, Ohio; 2 Department of Quantitative Health Sciences, Lerner Research Institute, Cleveland Clinic, Cleveland, Ohio; 3 Department of Molecular Cardiology, Lerner Research Institute, Cleveland Clinic, Cleveland, Ohio

**Keywords:** statins, thoracic aortic aneurysm surgery, postoperative renal failure

## Abstract

**Background** Patients undergoing surgery for thoracic aortic aneurysms receive statin therapy out of proportion to cardiovascular comorbidity. We sought to determine the prevalence of statin use among patients presenting for thoracic aortic aneurysm surgery and investigate its effect on outcomes.

**Methods** From January 1, 2005 to January 1, 2011, 1,839 consecutive patients underwent aortic replacement for degenerative thoracic aortic aneurysm at Cleveland Clinic. Of these, 771 (42%) were on statins preoperatively. Statin users (vs. nonstatin users) were older (65 ± 11 vs. 56 ± 16 years) and had more hypertension (78 vs. 59%). Propensity matching based on 56 preoperative variables other than lipid levels was used to compare outcomes among 570 matched patient pairs (74% of possible pairs).

**Results** Propensity-matched statin and nonstatin users were aged 64 ± 11 years, 394 (69%) versus 387 (68%) were male, and 437 (77%) versus 442 (78%) had ascending aortic aneurysms, respectively. Overall, 25% of patients were followed for more than 8.2 years and 10% for more than 10 years. Perioperative outcomes were similar, including hospital mortality (11 [1.9%] vs. 5 [0.88%]) and stroke (22 [3.9%] vs. 13 [2.3%]), but 16 statin users (2.8%) versus 5 nonstatin users (0.88%) required temporary dialysis after surgery ( *p* = 0.02). At 6 years, 3.7% of statin users versus 5.1% of nonstatin users ( *p* [log-rank] = 0.5) underwent further aortic surgery, and at 10 years, mortality was 25% in both groups ( *p* > 0.5).

**Conclusion** Patients presenting for thoracic aortic aneurysm surgery frequently receive unnecessary statins. Additionally, statin use was associated with more postoperative renal failure, but not less intermediate-term risk for aortic reintervention or all-cause mortality after surgery. Therefore, presence of a thoracic aortic aneurysm should not be considered an indication for statin therapy in the absence of well-established indications.

## Introduction

A major challenge in identifying medical therapies for thoracic aortic aneurysm has been its incompletely defined pathogenesis, although we know that hemodynamic, connective tissue, inflammatory, and genetic elements are linked to its development. Statins for abdominal aortic aneurysms have been shown to have beneficial effects in both human and animal studies 1 2 3 4 and are known to exert many pleiotropic, non–lipid-lowering effects, including modulation of the inflammatory system, improved endothelial function, antioxidant properties, and maintenance of atherosclerotic plaque stability, 2 in addition to their cardioprotective effects. 1 Nonetheless, there is widespread prophylactic use of statins in patients with thoracic aortic disease in the absence of well-established cardiovascular indications. Whether these same benefits can be extrapolated to thoracic aortic aneurysms is unknown. Therefore, we have investigated the prevalence of statin use among patients presenting for thoracic aortic aneurysm surgery and the cardiovascular factors associated with it, and we evaluated its possible effects on outcomes after aorta replacement.

## Materials and Methods

From January 1, 2005, to January 1, 2011, 1,839 consecutive adult patients underwent open surgical replacement of the thoracic aorta for degenerative thoracic aneurysm disease at Cleveland Clinic, a major referral center for this condition. Pediatric patients (age <18 years) and those with an acute or chronic dissection, pseudoaneurysm, mycotic aneurysm, or aortic rupture were not included. Of the 1,839 patients, 771 (42%) presented on statin therapy preoperatively (statin users), and 1,068 (58%) were not on statin therapy (nonstatin users). Typical cardiovascular indications for statin use (hyperlipidemia, coronary artery disease or prior myocardial infarction, peripheral or carotid artery disease, and prior stroke) were identified in 453 of the 771 statin users, but in 318 (41%), these indications were absent.

The aneurysm involved the aortic root in 403 patients (22%), ascending aorta in 1,407 (77%), aortic arch in 374 (20%), descending aorta in 160 (8.7%), and thoracoabdominal aorta in 160 (8.7%; Table 1 ). A connective tissue disorder was present in 133 (6.2%). The aortic valve was bicuspid or unicuspid in 814 (44%).

**Table 1:** Patient characteristics stratified by preoperative statin use in patients with thoracic aortic aneurysm: unmatched and propensity-matched groups

Characteristic	Unmatched patients					Propensity-matched patients				
	No preoperative statins ( *n* = 1,068)		Preoperative statins ( *n* = 771)		Standard difference	No preoperative statins ( *n* = 570)		Preoperative statins ( *n* = 570)		Standard difference
	*n* a	*n* (%) or mean ± SD	*n* a	*n* (%) or mean ± SD		*n* a	*n* (%) or mean ± SD	*n* a	*n* (%) or mean ± SD	
*Demographics* :										
Male	1,068	723 (68)	771	549 (71)	−7.6	570	387 (68)	570	394 (69)	−2.6
Age (y)	1,068	56 ± 16	771	65 ± 11	−67	570	64 ± 11	570	64 ± 11	−0.63
Body surface area (m ^2^ )	1,054	2.0 ± 0.29	764	2.1 ± 0.28	−7.0	561	2.1 ± 0.28	563	2.0 ± 0.28	3.6
*TAA details* :										
Location	1,068		771			570		570		
Root		269 (25)		134 (17)	19		104 (18)		104 (18)	0.0
Ascending		836 (78)		571 (74)	9.9		442 (78)		437 (77)	2.1
Arch		207 (19)		167 (22)	−5.6		119 (21)		121 (21)	−0.86
Descending		82 (7.7)		78 (10)	−8.6		56 (9.8)		56 (9.8)	0.0
Thoracoabdominal		59 (5.5)		101 (13)	−26		51 (8.9)		59 (10)	−4.8
Connective tissue disease	1,065	83 (7.8)	767	30 (3.9)	17	569	18 (3.2)	568	24 (4.2)	−5.6
*NYHAclass* :	1,067		768		−11	569		568		−1.4
I		590 (55)		375 (49)			293 (51)		283 (50)	
II		357 (33)		294 (38)			205 (36)		215 (38)	
III		113 (11)		88 (11)			67 (12)		65 (11)	
IV		7 (0.66)		11 (1.4)			4 (0.70)		5 (0.88)	
*Cardiac comorbidities* :										
Prior MI	1,068	74 (6.9)	771	142 (18)	−35	570	66 (12)	570	73 (13)	−3.8
Prior cardiac surgery	1,068	178 (17)	771	190 (25)	−20	570	107 (19)	570	117 (21)	−4.4
Bi- or unicuspid valve	1,066	516 (48)	767	298 (39)	19	569	255 (45)	568	250 (44)	1.6
*Noncardiac comorbidities* :										
Prior stroke	1,068	51 (4.8)	771	90 (12)	−25	570	41 (7.2)	570	51 (8.9)	−6.4
Peripheral arterial disease	1,068	66 (6.2)	771	108 (14)	−26	570	57 (10)	570	60 (11)	−1.7
Hypertension	1,068	631 (59)	771	605 (78)	−43	570	428 (75)	570	422 (74)	2.4
Pharmacologically treated diabetes	1,066	40 (3.8)	765	90 (12)	−30	568	36 (6.3)	566	45 (8.0)	−6.3
COPD	1,068	135 (13)	771	123 (16)	−9.5	570	85 (15)	570	84 (15)	0.48
Preoperative renal dialysis	1,068	40 (0.37)	771	1 (0.13)	4.9	570	3 (0.53)	570	1 (0.18)	5.9
Creatinine (mg/dL)	1,068	0.99 ± 0.47	770	1.0 ± 0.47	−11	570	1.0 ± 0.58	569	1.0 ± 0.52	1.8
*Cholesterol (mg/dL)* :										
Total b	846	187 ± 38	642	162 ± 32	72	452	187 ± 38	470	164 ± 33	63
HDL b	844	53 ± 17	642	51 ± 14	13	451	51 ± 17	470	51 ± 15	−0.063
LDL b	844	109 ± 32	642	87 ± 27	75	451	109 ± 32	470	89 ± 28	69
Triglycerides (mg/dL) b	845	132 ± 102	642	124 ± 69	8.8	451	134 ± 94	470	123 ± 72	13
*Preoperative medications* :										
Nonstatin lipid-lowering drug	1,060	49 (4.6)	769	81 (11)	−22	566	42 (7.4)	569	54 (9.5)	−7.4
ACE inhibitor	1,059	252 (24)	768	276 (36)	−27	566	181 (32)	569	183 (32)	−0.39
ARB	1,056	142 (13)	767	154 (20)	−18	565	106 (19)	569	95 (17)	5.4
Beta blocker	1,060	702 (66)	766	559 (73)	−15	564	393 (70)	566	397 (70)	−1.0
Calcium channel blocker b	489	98 (20)	334	112 (34)	−31	240	55 (23)	264	79 (30)	−16
*Preoperative echo measures* :										
Ejection fraction (%)	1,019	56 ± 7.6	723	55 ± 8.5	8.1	542	55 ± 7.9	538	55 ± 8.4	2.5
Aortic valve area (cm ^2^ ) b	275	1.1 ± 0.54	235	1.0 ± 0.47	18	154	1.0 ± 0.51	180	0.98 ± 0.45	12

Abbreviations: ACE, angiotensin-converting enzyme; ARB, angiotensin-II receptor blocker; COPD, chronic obstructive pulmonary disease; echo, echocardiogram; HDL, high-density lipoprotein; LDL, low-density lipoprotein; MI, myocardial infarction; SD, standard deviation; NYHA, New York Heart Association; TAA, thoracic aortic aneurysm.

a Patients with data available.

b Not included in propensity score.

The operation was performed through a full sternotomy in 1,543 patients (84%) and through a less-invasive incision in the remainder. 5 In 632 (34%), circulatory arrest was used. The most common procedure performed concomitantly with aorta replacement was aortic valve surgery in 1,387 (75%), repair in 386 (21%), and replacement in 1,001 (54%).

Patient characteristics and their preoperative imaging studies, medication use at presentation, and operative details were extracted for analysis from prospectively maintained registries and medical record review, all of which were approved for use in research by the Cleveland Clinic Institutional Review Board, with patient consent waived.

Endpoints included postoperative complications defined according to the Society of Thoracic Surgeons Adult Cardiac Surgery Database, operative mortality, any aortic event (dissection and rupture) or reintervention, and all-cause mortality during follow-up. Active anniversary follow-up occurred every 2 years, with 3,281 patient-years of active follow-up available for aortic events. Fifty percent of patients were followed-up for more than 1.5 years, 25% for more than 5.7 years, and 10% for more than 7.2 years for aortic events. Mortality follow-up data were supplemented by query of the Social Security Administration Death Master Index in October 2011, prior to the purging of 40% of the information 6 ; 50% were followed-up for more than 5.6 years, 25% for more than 8.2 years, and 10% for more than 10 years, with 10,157 patient-years of data available for analysis.

All analyses were performed using SAS statistical software version 9.4 (SAS Institute, Cary, NC) and R software version 3.5.0 (R Foundation for Statistical Computing, Vienna, Austria). Continuous variables are summarized as mean ± standard deviation or as equivalent 15th, 50th (median), and 85th percentiles when their distribution was skewed; comparisons were made using the Wilcoxon's rank-sum nonparametric test. Categorical variables are summarized by frequencies and percentages; comparisons were made using Chi-squared test, or Fisher's exact test if frequency was less than 5. Confidence intervals for time-related events are 68%, corresponding to ± 1 standard error.

There were substantial differences between statin users and nonusers at presentation for surgery ( Supplementary Fig. S1A ; available in the online version); thus, we developed a parsimonious multivariable logistic regression model (C-statistic 0.76) for statin use. For this, we used the machine-learning method, 7 whereby 1,000 bootstrap samples were analyzed by automated forward stepwise selection with replacement and a *p-* value for retention in the model of 0.05. A categorized set of variables was considered in the modeling, representing demographics, details of the aneurysm, cardiac comorbidity, chronic noncardiac comorbidities other than dyslipidemia and lipid levels, preoperative medications other than statins, and intended concomitant procedures, in addition to aortic replacement and date of operation ( Supplementary Appendix 1 ; available in the online version). These models were aggregated, and those appearing in 50% or more of the models (Breiman's median rule) were retained in the final model, with frequency of appearance characterized as variable reliability.

As noted in Table 1 , several variables examined in multivariable analyses had missing values, generally at a frequency, we consider missing at random. We used five-fold multiple imputation with the Markov chain Monte Carlo technique to impute missing values and construct final multivariable models. 8

Having established the parsimonious model for statin use, we added other variables representing demographics, symptoms, cardiac and noncardiac comorbidities, and intended procedure variables that might be related to unrecorded selection factors, producing a saturated model consisting of the 56 variables ( Supplementary Appendix 1 ; available in the online version). This model had a C-statistic of 0.78. A propensity score was calculated for each patient by solving the saturated model for the probability of being in the statin user group. Then, using the propensity score, statin users were matched to nonstatin users by a greedy matching strategy. This yielded 570 pairs, 74% of possible matches ( Supplementary Fig. S1A ; available in the online version). Standardized differences were well within the ± 10% range, indicating well-matched cohorts ( Supplementary Fig. S1B ; available in the online version).

Freedom from aortic events was assessed nonparametrically using the Kaplan–Meier method; comparisons of statin users and nonusers were made using the log-rank test. Parametric estimates of survival were assessed by a multiphase hazard model. 9

## Results

The most commonly prescribed statins were atorvastatin (343/769, 45%) and simvastatin (254/769, 33%; Table 2 ; Supplementary Table S1 [available in the online version]). Compared with nonusers, statin users were older, had more hypertension but lower cholesterol levels ( Supplementary Fig. S1B ; available in the online version), and had more distal thoracic aortic disease ( Table 1 ). Statin users also had more carotid artery disease and were more likely to have experienced preoperative cardiovascular events. Nevertheless, because cardiac catheterization was performed routinely as standard of care before surgery for thoracic aortic aneurysms, we were able to document that 318 statin users (41%) did not have coronary artery disease (no epicardial coronary artery with 50% or more stenosis), peripheral arterial disease, or carotid artery disease, which we interpreted as not having a cardiovascular indication for statins. Effectiveness of statins was equally evident in statin users with or without a cardiovascular indication for statins ( Supplementary Fig. S2 ; available in the online version). By multivariable analysis, older age, ischemic heart disease, use of an angiotensin-converting enzyme inhibitor or angiotensin-receptor blocker, distal thoracic aorta disease, diabetes, and more recent date of presentation for surgery were among the highly reliable variables associated with statin use, exclusive of lipid levels ( Table 3 ).

**Table 2:** Procedure details stratified by preoperative statin use for unmatched and propensity-matched groups

Variable	Unmatched patients			Propensity-matched patients		
	No preoperative statins ( *n* = 1,068)	Preoperative statins ( *n* = 771)	Standard difference	No preoperative statins ( *n* = 570)	Preoperative statins ( *n* = 570)	Standard difference
	*n* (%) or mean ± SD	*n* (%) or mean ± SD		*n* (%) or mean ± SD	*n* (%) or mean ± SD	
*Procedure* :						
Coronary artery bypass grafting	130 (12)	235 (30)	−46	118 (21)	130 (23)	−5.1
Aortic valve surgery	851 (80)	536 (70)	24	416 (73)	416 (73)	0.0
Mitral or tricuspid valve surgery	71 (6.6)	53 (6.9)	−0.90	42 (7.4)	40 (7.0)	1.4
Aortic root, ascending aorta, arch replacement	1,018 (95)	703 (91)	17	524 (92)	527 (92)	−2.0
Descending thoracic aorta grafting	81 (7.6)	102 (13)	−19	66 (12)	64 (11)	1.1
*Support* :						
Cardiopulmonary bypass time (min)	103 ± 46	110 ± 54	−14	103 ± 48	105 ± 52	−5.5
Circulatory arrest	357 (33)	275 (36)	−4.7	197 (35)	199 (35)	−0.74
Circulatory arrest time (min) a	17 ± 11	19 ± 13	−10	18 ± 12	18 ± 12	−0.74

Abbreviations: SD, standard deviation.

a Unmatched group: data available for 357 patients in the nonstatin cohort and 275 in the statin cohort. Propensity-matched group: data available for 197 patients in the nonstatin cohort and 199 in the stain cohort.

**Table 3:** Patient factors associated with preoperative statin use versus nonuse

Factor	Coefficient ± standard error	*p* -Value	Reliability (%) a
*Higher likelihood of preoperative statin use* :			
Male	0.38 ± 0.12	**0.002**	51
Older age			100
Age b	6.4 ± 0.96	**<0.0001**	–
Age c	−1.2 ± 0.24	**<0.0001**	–
Prior myocardial infarction	0.49 ± 0.17	**0.005**	61
Prior cardiac surgery	0.39 ± 0.14	**0.004**	75
Left anterior descending coronary artery system disease (≥50% stenosis)	0.85 ± 0.16	**<0.0001**	98
Pharmacologically treated diabetes	0.71 ± 0.21	**0.0009**	79
Prior stroke	0.57 ± 0.20	**0.006**	76
Preoperative angiotensin-converting enzyme inhibitor	0.48 ± 0.12	**<0.0001**	86
Preoperative nonstatin lipid-lowering medication	0.49 ± 0.21	**0.02**	56
Preoperative angiotensin II receptor blocker	0.46 ± 0.15	**0.002**	72
Thoracoabdominal thoracic aortic aneurysm	0.46 ± 0.19	**0.02**	83
More recent date of operation d	0.25 ± 0.064	**<0.0001**	95
*Higher likelihood of no preoperative statin use* :			
Greater preoperative left ventricular end-diastolic volume indexed to body surface area	−0.010 ± 0.0024	**<0.0001**	58

a Percentage of times variable or cluster of variables appeared in 1,000 bootstrap models.

b Ln(age), natural logarithmic transformation.

c Exp(age/50), exponential transformation.

d Ln(January 1, 2005 to date of index surgery), natural logarithmic transformation.

Matched statin users had more perioperative complications than matched nonstatin users; however, renal failure requiring temporary dialysis was the only statistically significant one ( Table 4 ), occurring in 16 statin users (2.8%) and 5 nonusers (0.88%; *p* = 0.02). It was more common in patients with extensive aortic disease (12 thoracoabdominal repairs, 4 arch replacements, and 4 ascending aorta replacements).

**Table 4:** Acute outcomes in matched cohort

Outcome	No preoperative statins( *n* = 570)		Preoperative statins( *n* = 570)		*p* -Value
	*n* a	*n* (%) or 15th/50th/85th percentiles	*n* a	*n* (%) or 15th/50th/85th percentiles	
Hospital death	570	5 (0.88)	570	11 (1.9)	0.13
Permanent stroke	570	13 (2.3)	570	22 (3.9)	0.12
Reintervention for bleeding or tamponade	570	24 (4.2)	570	32 (5.6)	0.3
New-onset atrial fibrillation b	531	178 (34)	529	183 (35)	0.7
Renal failure c	570	21 (3.7)	570	34 (6.0)	0.07
New-onset renal failure requiring dialysis b	567	5 (0.88)	569	16 (2.8)	**0.02**
Prolonged ventilation (>24 hours)	570	98 (17)	570	116 (20)	0.17
Myocardial infarction	570	2 (0.35)	570	0 (0)	0.5
Length of stay	569		570		
Intensive care unit (h)		23/40/139		22/45/136	0.5
Operative (d)		5.0/7.1/13		5.1/7.1/13	0.4

a Patients with data available.

b “New-onset” excludes patients who had atrial fibrillation or dialysis preoperatively and those whose preoperative status was unknown and did not have an outcome.

c Renal failure was defined as an increase in serum creatinine levels of ≥4 mg/dL (176.8 mmol/L), a ≥50% increase in serum creatinine levels over the baseline preoperative value, or a new requirement for dialysis.

**Fig. 1:**
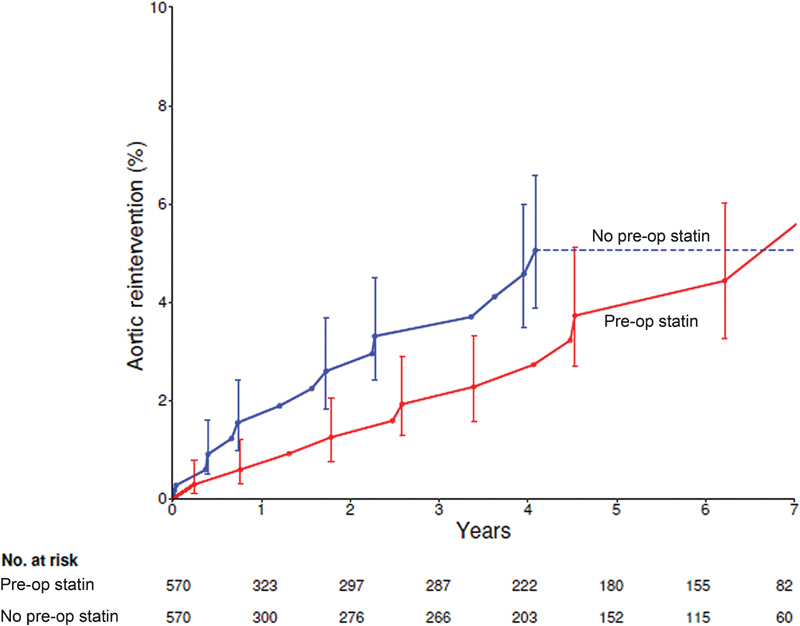
Aortic reinterventions in propensity-matched cohorts after surgery (time 0) for thoracic aortic aneurysm. Each symbol represents a reintervention positioned on the vertical axis by the Kaplan–Meier estimator, and vertical bars are 68% confidence limits equivalent to ± 1 standard error. Red lines and circles, preoperative (pre-op) statin use group; blue lines and circles, no preoperative (pre-op) statin use group.

Among matched patients, there were 12 reinterventions for thoracic aorta disease in statin users and 14 in nonusers. At 1, 5, and 7 years, aortic events occurred in 0.60, 3.7, and 4.4% of statin users and 1.6, 5.1, and 5.1% of nonstatin users, respectively ( *p* [log-rank] = 0.5; Fig. 1 ; Supplementary Fig. S3 ; available in the online version). Reinterventions included reoperative aortic root surgery and ascending aorta replacement with an allograft in 15, thoracic endovascular aorta repair in 7, reoperative ascending aorta replacement in 3, and redo open thoracoabdominal repair in 1.

**Fig. 2:**
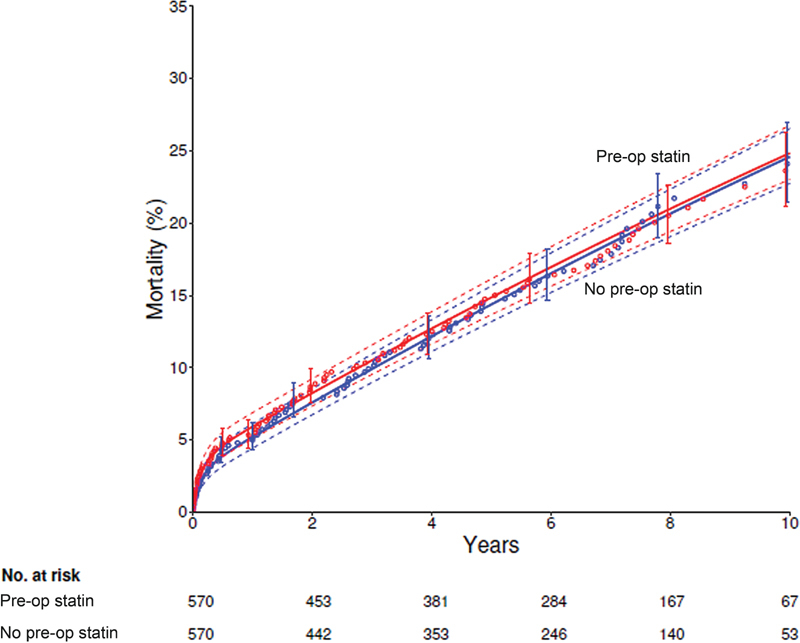
Mortality in propensity-matched cohorts after surgery for thoracic aortic aneurysm. Each symbol represents a death positioned on the vertical axis by the Kaplan–Meier estimator, and vertical bars are 68% confidence limits equivalent to ± 1 standard error. Solid lines are parametric survival estimates enclosed within dashed 68% confidence bands. Red curves and circles = preoperative (pre-op) statin use group; blue curves and circles = no preoperative (pre-op) statin use group.

Among matched patients, there were 101 deaths in statin users versus 93 in nonusers. At 1, 5, and 10 years, mortality was 5.9, 15, and 25% among statin users versus 5.2, 14, and 25% among nonstatin users, respectively (early hazard phase *p* = 0.5, constant hazard phase *p* = 0.8; Fig. 2 ; Supplementary Fig. S4 ; available in the online version).

## Discussion

Statin users represent a high cardiovascular risk group, and their outcomes were distinctly worse than those of nonstatin users. These worse outcomes can be attributed to higher risk patient profiles and not to statin use per se, as evidenced by mostly similar outcomes in propensity-matched cohorts. However, the requirement for temporary renal dialysis for acute postoperative renal failure was more common in propensity-matched statin users than nonusers.

Statin use for abdominal aortic aneurysms has been shown to have beneficial effects in both human 10 11 12 13 14 and animal 15 studies. Statins reduce abdominal aortic aneurysm formation in mice, 15 and in humans, 10 11 they have been associated with decreased rates of aortic aneurysm growth and rupture. 11 12 13 14 A proposed mechanism for this effect is believed to involve inhibition of metallopeptidase-9 production by activated neutrophils and macrophages within aortic tissue. 10 15 16 Inhibition of the endoplasmic reticulum stress-signaling pathway by statins may also contribute to preventing aortic aneurysm progression. 17

Thoracic aortic disease differs from abdominal aortic disease, however. The study by Angeloni and colleagues 12 of the ascending aorta growth rate showed that over a 3-year period, statins slowed growth, but only by a millimeter on average, the clinical significance of which is suspected. Jovin and colleagues 18 found that statin users underwent operation less often over time than nonstatin users in their long-term study of statin use in patients with thoracic aortic aneurysm. Similarly, Stein and colleagues 19 reported that a much larger number of patients on statins were free of dissection, aortic rupture, or death than nonstatin users, although their study did not account for other differences, such as use of other medications that might have contributed to their findings.

Perioperative statin therapy in the cardiac surgery setting has been associated with mixed effects with regard to safety, and specifically with regard to acute kidney injury. Verdoodt and colleagues 20 review arguments in favor of statin use, noting, for example, that a meta-analysis including nearly 60,000 patients showed 13% lower postoperative acute kidney injury after cardiac surgery. 21 Zhao and colleagues 22 published their meta-analysis of eight randomized controlled trials and found a nonsignificant 9% increased risk of acute kidney injury requiring dialysis after cardiac surgery. Putzu and colleagues, 23 however, found that perioperative statins were associated with a higher occurrence of acute kidney injury after cardiac surgery in their meta-analysis of 11 randomized controlled trials, showing a relative risk of 15% higher than that of nonstatin users. Zheng and colleagues 24 randomized patients to a placebo or rosuvastatin of 20 mg daily for up to 8 days before cardiac surgery (87% coronary artery bypass grafting) and for 5 days thereafter. Any grade of acute kidney injury at 48 hours after surgery occurred in 25% of statin users and 19% of nonusers ( *p* = 0.005). These trials differed from our study in that they involved perioperative statin administration, and the cohort consisted largely of patients undergoing coronary artery bypass grafting, whereas our patients were on chronic statins preoperatively and all underwent thoracic aorta surgery.

In contrast to our findings, Tyerman and colleagues, 25 using the 19-institution Virginia Cardiac Services Quality Initiative database, reported no statistically significant differences in perioperative outcome among statin users and nonusers, with dialysis for acute renal failure in 8 of 386 matched pairs among statin users (2.1%) versus 13 (3.4%) among nonusers undergoing ascending aorta replacement.

Given this information on the effectiveness and safety of statins, we conclude that with lack of clear benefit and possible adverse effect on renal function, it is prudent not to recommend statin therapy in the absence of a cardiovascular indication.

## Limitation

The major limitation of this study is that all patients presented with surgical indication for aorta replacement. As such, we were able to study only the effects of statin use on postoperative complications, aortic interventions after aorta replacement, and mortality. Thus, this was a safety study. We do not know whether statin use was effective in slowing the growth of the aneurysm or if it prevented dissection or rupture. Ours is also a single-institution observational study of consecutive patients. Statins were not randomly prescribed, so we used propensity-score matching to make a fair comparison of average treatment effect.

## Conclusion

In conclusion, the potential benefit of chronic statin therapy in slowing dilatation of the thoracic aorta has not been established. When counseling patients with thoracic aortic disease who use statins, physicians should ascertain whether there is an established cardiovascular indication. From our study and others, the mere presence of a thoracic aortic aneurysm should not serve as an indication for statin therapy because there is no clear benefit and a possible perioperative risk of acute kidney injury. Consideration should be given to discontinuing statins in such patients if the indication is not hyperlipidemia or cardiovascular disease.
