# Supplementary material for: Statin Therapy in Patients Undergoing Thoracic Aorta Replacement for Aortic Aneurysms
Source: Aorta (Stamford). 2021 Nov 8;9(4):147–54. doi: 10.1055/s-0041-1730296 (PMC8642073; doi:10.1055/s-0041-1730296)
Supplement: Supplementary file 1 — Supplementary Material [file 10-1055-s-0041-1730296-s200053.pdf]

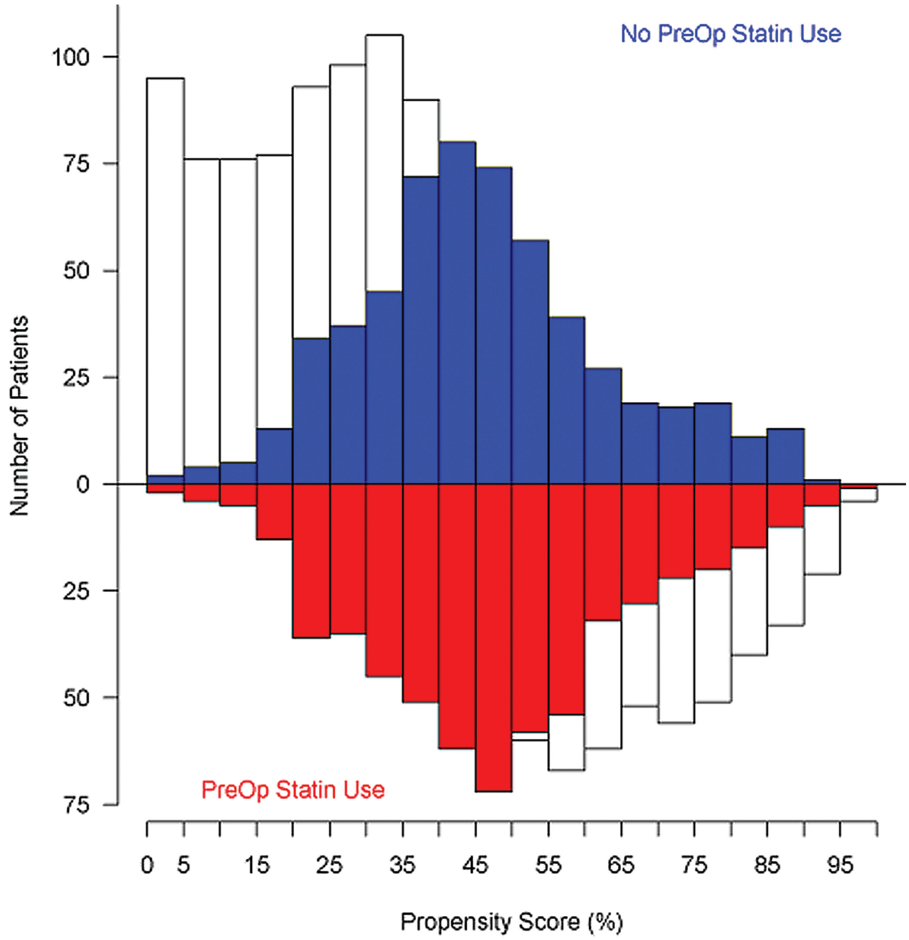

Supplementary Fig. S1 Quality of propensity matching of patients taking or not taking preoperative (pre-op) statins who underwent aortic replacement for degenerative thoracic aortic aneurysm. **(A)** Mirrored histogram of distribution of propensity scores between groups. Shaded areas indicate matched patient pairs, and clear bars represent unmatched cases. Quality of propensity matching of patients taking or not taking preoperative (pre-op) statins who underwent aortic replacement for degenerative thoracic aortic aneurysm. **(B)** Standardized differences of selected variables before and after matching, illustrating good matching (values within ± 10%). Values on the horizontal axis represent percent standardized difference between groups. Red triangles represent standardized differences before matching, green squares standardized differences after matching.

 © 2021. The Author(s).

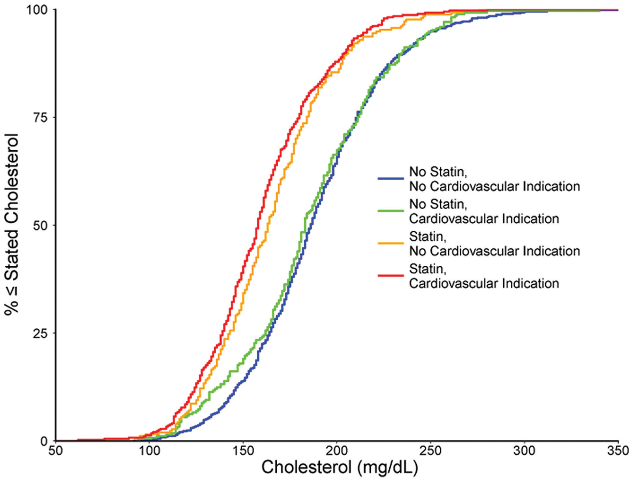

Supplementary Fig. S2 Cumulative distribution of preoperative cholesterol levels in patients on statins and not on them before aortic replacement for thoracic aortic aneurysm, stratified by whether or not they had a cardiovascular indication for a statin. Left-most red curve is for statin users with a cardiovascular indication; yellow curve next to it is for statin users without a cardiovascular indication; next green curve is for non-statin users with a cardiovascular indication; and right-most blue curve is for non-statin users with no cardiovascular indication. Solid lines represent the cumulative distribution of cholesterol levels by statin and cardiovascular indication.

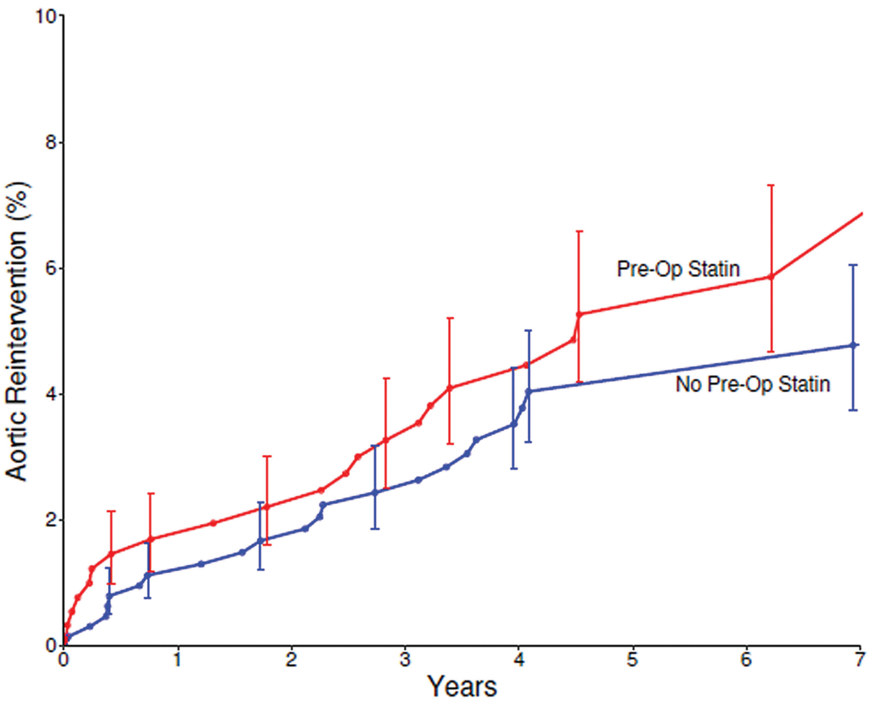

No. at risk								
Pre-Op Statin	771	410	376	359	269	216	181	94
No Pre-Op Statin	1,068	589	524	500	380	289	214	126

Supplementary Fig. S3 Aortic reinterventions after surgery (time 0) for thoracic aortic aneurysm. These are unmatched, non–risk-adjusted groups. Each symbol represents a reintervention positioned on the vertical axis by the Kaplan-Meier estimator, and vertical bars are confidence limits equivalent to ±1 standard error. Red curves and filled circles are for the preoperative (Pre-op) statin use group, and blue curves and filled circles are for the no preoperative statin use group.

 © 2021. The Author(s).

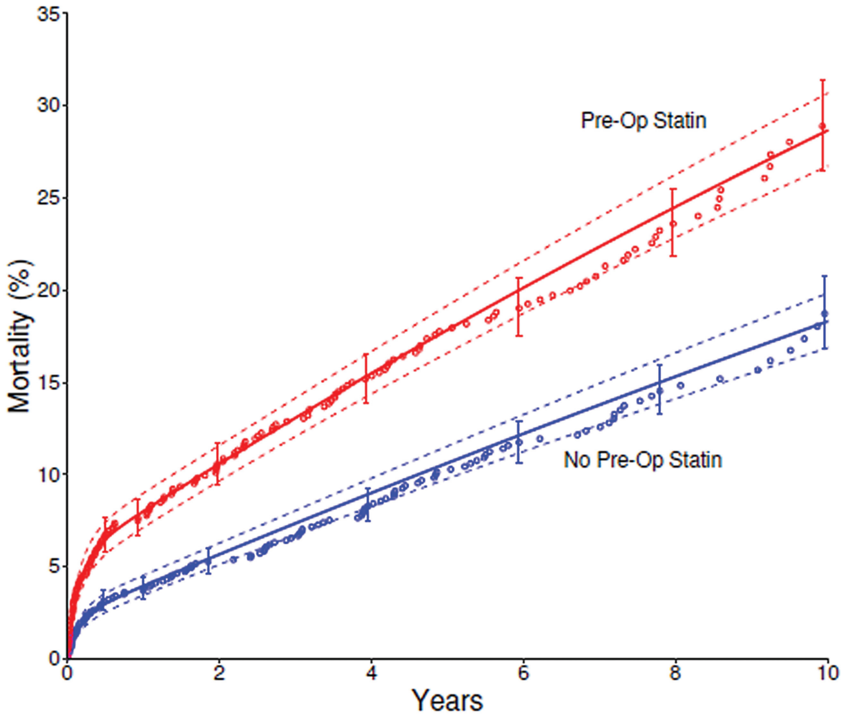

No. at risk						
Pre-Op Statin	771	593	486	361	208	81
No Pre-Op Statin	1,068	865	695	492	288	115

Supplementary Fig. S4 Mortality after surgery for thoracic aortic aneurysm. These are unmatched, non–risk-adjusted groups, with separation of curves reflecting differences in risk profile of the 2 groups. Each symbol represents a death positioned on the vertical axis by the Kaplan-Meier estimator, and vertical bars are 68% confidence limits equivalent to ± 1 standard error. Solid lines are parametric survival estimates enclosed within dashed 68% confidence bands. Red curves and filled circles = preoperative (Pre-op) statin use group, and blue curves and filled circles = no preoperative statin use group.

Supplementary Table S1 Statins prescribed preoperatively ($n = 769$)

Statin	*n* (%)
Atorvastatin	343 (45)
Fluvastatin	5 (0.65)
Lovastatin	33 (4.3)
Mevastatin	6 (0.78)
Pravastatin	57 (7.4)
Rosuvastatin	71 (9.2)
Simvastatin	254 (33)

Supplementary Appendix

Variables considered in analyses[a]

Demographics

Age (y),[a] weight (kg), height (cm), weight/height ratio, body surface area (m^2), body mass index (kg/m^2),[a] sex,[a] and race (white,[a] black).

 © 2021. The Author(s).

Symptoms
New York Heart Association functional class (I–IV).[a]

Details of Thoracic Aortic Aneurysm
Thoracic aortic aneurysm (TAA) location (root,[a] ascending,[a] arch,[a] descending,[a] thoracoabdominal[a]), connective tissue disease,[a] and bicuspid aortic valve.[a]

Ventricular Dysfunction
Prior myocardial infarction.[a]

Cardiac Comorbidity
Atrial fibrillation,[a] complete heart block or pacer,[a] ventricular arrhythmia,[a] prior cardiac operation,[a] heart failure,[a] endocarditis,[a] and number of cardiac operations.

Noncardiac Comorbidity
Peripheral arterial disease,[a] carotid disease,[a] hypertension,[a] pharmacologically treated diabetes,[a] non–insulin-treated diabetes, chronic obstructive pulmonary disease,[a] history of smoking,[a] prior stroke,[a] creatinine (mg/dL),[a] blood urea nitrogen (mg/dL),[a] bilirubin (mg/dL),[a] creatinine clearance (mL/min), glomerular filtration rate (mL/min/1.73 m^2), and hematocrit (%).[a]

Preoperative Medications
Nonstatin lipid-lowering drug,[a] angiotensin-converting enzyme inhibitor,[a] angiotensin-II receptor blocker,[a] and β-blocker.[a]

Coronary Artery Disease
System disease greater than 50% stenosis (left anterior descending coronary artery,[a] left circumflex coronary artery,[a] left main trunk,[a] right coronary artery), and number of systems with greater than 50% stenosis.[a]

Preoperative Echocardiographic Measures

Left ventricular structure: inner diameter in diastole (cm),[a] inner diameter in systole (cm),[a] diastolic volume (mL),[a] systolic volume (mL),[a] diastolic volume index (mL/m^2),[a] systolic volume index (mL/m^2),[a] and calculated left ventricular relative wall thickness.[a]
Left ventricular function: fractional shortening, ejection fraction (%),[a] and calculated left ventricular ejection fraction.
Left ventricular mass: mass (g),[a] mass index (g/m^2), posterior wall thickness (cm),[a] and septal thickness (cm).[a]
Left atrial size: left atrial diameter (cm),[a] calculated unscaled left atrial volume (cm^3),[a] unscaled left atrial volume index (cm^3/m^2), and left atrial size $\geq$6 cm.[a]

Experience
Date of operation (years since January 1, 2005).[a]

Procedure
Coronary artery bypass grafting,[a] aortic valve procedure,[a] mitral valve procedure,[a] and tricuspid valve procedure.[a]

[a]*Denotes variables used in the propensity model.*

 © 2021. The Author(s).
